# Structure and Evolution of Glycogen Branching Enzyme N-Termini From Bacteria

**DOI:** 10.3389/fmicb.2018.03354

**Published:** 2019-01-14

**Authors:** Liang Wang, Qinghua Liu, Junfeng Hu, James Asenso, Michael J. Wise, Xiang Wu, Chao Ma, Xiuqing Chen, Jianye Yang, Daoquan Tang

**Affiliations:** ^1^Department of Bioinformatics, School of Medical Informatics, Xuzhou Medical University, Xuzhou, China; ^2^Jiangsu Key Laboratory of New Drug Research and Clinical Pharmacy, School of Pharmacy, Xuzhou Medical University, Xuzhou, China; ^3^Computer Science and Software Engineering, University of Western Australia, Perth, WA, Australia; ^4^The Marshall Centre for Infectious Diseases Research and Training, University of Western Australia, Perth, WA, Australia; ^5^Center for Experimental Animals, Xuzhou Medical University, Xuzhou, China

**Keywords:** glycogen, branching enzyme, N1 domain, CBM48, average chain length, durability

## Abstract

In bacteria, glycogen plays important roles in carbon and energy storage. Its structure has recently been linked with bacterial environmental durability. Among the essential genes for bacterial glycogen metabolism, the *glgB*-encoded branching enzyme GBE plays an essential role in forming α-1,6-glycosidic branching points, and determines the unique branching patterns in glycogen. Previously, evolutionary analysis of a small sets of GBEs based on their N-terminal domain organization revealed that two types of GBEs might exist: (1) Type 1 GBE with both N1 and N2 (also known as CBM48) domains and (2) Type 2 GBE with only the N2 domain. In this study, we initially analyzed N-terminal domains of 169 manually reviewed bacterial GBEs based on hidden Markov models. A previously unreported group of GBEs (Type 3) with around 100 amino acids ahead of the N1 domains was identified. Phylogenetic analysis found clustered patterns of GBE types in certain bacterial phyla, with the shorter, Type 2 GBEs predominantly found in Gram-positive species, while the longer Type 1 GBEs are found in Gram-negative species. Several *in vitro* studies have linked N1 domain with transfer of short oligosaccharide chains during glycogen formation, which could lead to small and compact glycogen structures. Compact glycogen degrades more slowly and, as a result, may serve as a durable energy reserve, contributing to the enhanced environmental persistence for bacteria. We were therefore interested in classifying GBEs based on their N-terminal domain via large-scale sequence analysis. In addition, we set to understand the evolutionary patterns of different GBEs through phylogenetic analysis at species and sequence levels. Three-dimensional modeling of GBE N-termini was also performed for structural comparisons. A further study of 9,387 GBE sequences identified 147 GBEs that might belong to a possibly novel group of Type 3 GBE, most of which fall into the phylum of *Actinobacteria*. We also attempted to correlate glycogen average chain length (ACL) with GBE types. However, no significant conclusions were drawn due to limited data availability. In sum, our study systematically investigated bacterial GBEs in terms of domain organizations from evolutionary point of view, which provides guidance for further experimental study of GBE N-terminal functions in glycogen structure and bacterial physiology.

## Introduction

Glycogen is a widespread water-soluble homogeneous polysaccharide with α-1,4-glycosidic bonds in linear chains and α-1,6-glycosidic linkages at the branching points (Wang and Wise, 2011). As a carbon and energy reserve, glycogen has so far been identified in archaea, bacteria and heterotrophic eukaryotes, etc. (Ball et al., 2015). It is usually accumulated under limited growth conditions with excessive carbon sources but deficient in other nutrients (Wilson et al., 2010). During unfavorable times, glycogen as carbon and energy reserves is then broken down to support bacterial long-term survival (Wang and Wise, 2011). A seminal study on 55 bacterial genomes concluded that loss of glycogen metabolism could be correlated with a parasitic lifestyle (Henrissat et al., 2002). Further study based on 1,202 bacterial genome analyses confirmed that bacteria harboring the classic glycogen metabolism genes occupy more diverse niches (Wang and Wise, 2011). Thus, glycogen is a potential indicator for bacterial lifestyle. A variety of experimental studies also linked glycogen with bacterial energy metabolism (Wilson et al., 2010; Chandra et al., 2011), environmental persistence (McMeechan et al., 2005; Wang and Wise, 2011), dormancy (Sawers, 2016; Klotz and Forchhammer, 2017), and virulence (Jones et al., 2008; Gupta et al., 2017), etc., suggesting that glycogen plays a central role in bacterial physiology through widespread connections among abundant cellular pathways (Wilson et al., 2010; Chandra et al., 2011). Recently, glycogen structure draws more attention for its potential functions in bacterial physiology (Wang and Wise, 2011; Klotz and Forchhammer, 2017). The uniquely branched structure of glycogen in bacteria is normally due to a group of coordinated enzymes: ADP-glucose pyrophosphorylase (GlgC), glycogen synthase (GlgA), glycogen branching enzyme (GlgB or GBE), glycogen phosphorylase (GlgP), and glycogen debranching enzyme (GlgX) (Wilson et al., 2010; Wang and Wise, 2011). GlgC, GlgA, and GBE are involved in glycogen synthesis processes, while GlgP and GlgX function as degradation enzymes (Wang and Wise, 2011). Specifically, glucose-1-phosphates are first catalyzed into ADP-glucoses by GlgC, which are then used as building blocks for synthesizing linear α-1,4-polyglucan with the assistance of GlgA (Wilson et al., 2010). In contrast, eukaryotes utilize UDP-glucose for glycogen synthesis (Wilson et al., 2010). GBE is mainly responsible for formation of the characteristic branching pattern in glycogen. Take GBE in *E. coli* for example. α-1,4-glycosidic bonds in linear chains are broken first and short oligosaccharides no less then six glucosyl residues, with eight or more glucosyl residues preferred, are then transferred to neighboring or the same chains to form α-1,6-glycosidic bonds linked branches (Binderup et al., 2000; Wang et al., 2015).

In bacteria, average chain length (ACL) of glycogen is reported to be around 8–12 glucosyl residues (degree of polymerization, known as d.p.) and the molecular size is estimated to be about 10^7^ to 10^8^ Daltons, similar to animal glycogen (Wilson et al., 2010). However, there are many exceptions. For example, glycogen ACL in *Thermus thermophilus* is only 7 d.p., while in *Clostridium botulinum* it reaches to 17 d.p. (Wang and Wise, 2011). A study by Wang and Wise (2011) extended the range of glycogen ACL to between 7 and 21 d.p. In contrast, glycogen structure in animals is normally consistent and ACL is less than or equal to 13 d.p. (Wang and Wise, 2011). This indicated that (a) GBE has distinct and varied branching specificity in bacteria than in animals, and (b) bacteria glycogen structure is more variable than animal glycogen structure in terms of ACL (Feng et al., 2015). As for degradation of glycogen, GlgP repeatedly removes non-reducing terminal glucosyl residues as glucose-1-phosphates until four glucosyl residues from the branch points remain, and then GlgX works on the short chains by truncating the α-1,6-glycosidic bonds (Dauvillee et al., 2005; Alonso-Casajus et al., 2006). The truncated maltotetraose was then employed by MalQ and MalP for glycogen synthesis and maltodextrin degradation, respectively (Park et al., 2011). It is noteworthy that GlgX in human is a bifunctional enzyme with both α-1,4-glucanotransferase and α-1,6-glucosidase activities, which transfers three residues from the GlgP-processed branched chain to a nearby or the same chain, leaving only one glycosyl residue removed later by α-1,6-glucosidase activity (Zhai et al., 2016). Although each enzyme of glycogen metabolism has a unique function, abundant studies show that these enzymes work together to maintain a balance between glycogen biosynthesis and degradation processes, which might lead to different glycogen primary, secondary and tertiary conformations in bacteria (Wang and Wise, 2011; Feng et al., 2015). Recent study also proposed that cytoplasmic α-amylase or an unknown maltose phosphorylase could be responsible for glycogen degradation (Strydom et al., 2017). In addition, glycogen structure has also been linked with blood glucose control in type 2 diabetes mellitus (T2DM) (Sullivan et al., 2011). Thus, a clear understanding of the regulation of glycogen structure in bacteria might shed some light on the structural abnormalities of glycogen and T2DM in higher organisms.

Among all glycogen-related enzymes, GBE (EC 2.4.1.18) is unquestionably one of the most important, and is the determining factor for glycogen structure (Wang and Wise, 2011; Wang et al., 2015). It is reported to be highly conserved in all forms of life (Zmasek and Godzik, 2014). Bacterial GBE belongs to GH13 family of glucosyl hydrolase (α-amylase family) and has three common domains, that is, CBM48 at N-terminus, central (β/α)_8_-barrel domain (α-amylase) containing active sites, and a conserved C-terminal β-sandwich domain (C-terminus) (Feng et al., 2015). Another branching enzyme with (β/α)_7_-barrel at the N-terminus as a catalytic domain belongs to GH57 and was first discovered in a hyperthermophilic archaeal species *Thermococcus kodakaraensis* KOD1 (Murakami et al., 2006; Blesak and Janecek, 2012). Although GBE belonging to GH57 has been detected in several bacterial phyla such as *Firmicutes*, *Actinobacteria*, and *Cyanobacteria*, most of GH57 branching enzymes are poorly chemically described (Murakami et al., 2006; Blesak and Janecek, 2012; Zmasek and Godzik, 2014). In this study, only bacterial branching enzyme is investigated and all GBEs belong to GH13 family by default, unless otherwise stated.

The initially failed attempt of crystallizing full-length GBE drew researchers’ attentions to its extended N-terminus in several bacteria, such as *Escherichia coli* and *Deinococcus geothermalis*, *etc.* (Binderup et al., 2000; Hilden et al., 2000; Palomo et al., 2009; Pal et al., 2010). Recently, a couple of studies reported the full-length structure of GBEs in *Mycobacterium tuberculosis* and *Cyanothece* sp. ATCC 51142, which increases our understanding of spatial structures and physiological functions of GBEs in bacteria (Pal et al., 2010; Hayashi et al., 2017). Crystal structure of human GBE1 was also solved recently and provided more details about glycogen storage disorder Type IV (GSDIV) (Froese et al., 2015). So far, it has been proposed that there are two types of GBEs, Type 1 and Type 2, in bacteria according to the N-terminal differences (Hilden et al., 2000; Leggio et al., 2002; Devillers et al., 2003; Palomo et al., 2009; Jo et al., 2015; Wang et al., 2015). Type 1 GBE has an experimentally confirmed domain N2 and a 100 aa region, termed N1 domain, ahead of it, while type 2 GBE has N2 domain only (Leggio et al., 2002). N1 domain of GBE is proposed to link with branching patterns in bacterial glycogen synthesis (Devillers et al., 2003; Palomo et al., 2009; Jo et al., 2015). Truncation of GBE N1 domain showed a reduction of 50–60% full-length enzymatic activity (Hilden et al., 2000), while progressively shortening N-terminus of type 1 GBE up to 112 aa in *E. coli* led to gradual increase in the length of transferred chains (Devillers et al., 2003). However, Devillers et al. (2003) used engineered GBE digesting the linear polysaccharide amylose *in vitro*, which, to some degree, does not reflect *in vivo* situation in bacteria. A couple of experiments involving swapping GBE N-terminal domains proved that N1 domain is correlated with short oligosaccharide chain transfer, and N2 domain could also have similar functions (Kuriki et al., 1997; Palomo et al., 2009; Jo et al., 2015). In a more recent study, Wang et al. (2015) showed that *in situ* truncation of GBE N1 domain in *E. coli* actually leads to a shift of chain length distributions toward longer region. However, no length-dependent correlation was identified (Wang et al., 2015). A similar study conducted in *Mycobacterium tuberculosis* suggested that N1-truncated GBE had 50% less activity and had the same substrate binding ability as full-length GBE when starch was used as a substrate (Pal et al., 2010). In parallel to the biophysical characterization of GBE, another study linked short glycogen ACL with bacteria environmental persistence due to the slow degradation property (Wang and Wise, 2011). Although the function of N-terminus in GBE is still controversial, the presence of N1 domain could be a determining factor of glycogen structure based on above-mentioned evidences. However, more experimental data are needed to establish any potential correlations. It is also noteworthy that GH13 GBEs in CAZy database are divided into GH13_8 (eukaryotic GBEs) and GH13_9 (prokaryotic GBEs), which are based on similarity and variation of α-amylase domain and should not be confused with our GBE classification based on N-terminal structures (Lombard et al., 2014; Froese et al., 2015).

**FIGURE 1:**
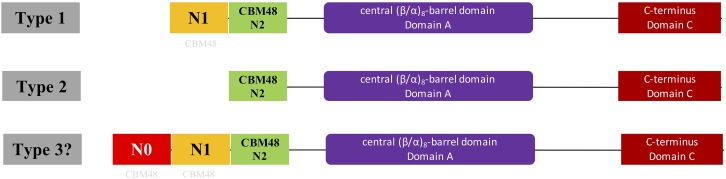
Illustration of domain organizations of different types of bacterial glycogen branching enzymes based on N-terminal organization. Type 1 and 2 GBEs were previously confirmed through theoretical and experimental studies while type 3 GBE has a presumed N0 domain longer than 100 aa ahead of N1 domain and is firstly proposed in this study, which might represent a new class of GBEs. However, genuineness of the existence of Type 3 GBE is still in question and requires further experimental exploration. N1 and N2 are around 100 aa while N0 is greater than 100 aa. N0 and N1 domains were suggested to be distant relatives to the standard CBM48 domain (N2 domain) found in Pfam and were labeled with light gray CBM48.

Glycogen structure has recently been proposed to play important roles in bacterial durability (Wang and Wise, 2011). In addition, the N-terminus of GBE has been confirmed experimentally to control glycogen branching degree and ACL (Wang et al., 2015). Thus, N-terminus centered classification of bacterial GlgB might promote our understanding of bacterial durability. In this study, we initially collected 169 manually reviewed bacterial GBE protein sequences from UniProt database for N-terminal analysis. These belonged to 161 bacterial species due to *glgB* gene duplications in eight bacteria (Supplementary Table S1). N-terminal domain structures were analyzed using Hidden Markov Model screening (Eddy, 2004). Phylogenetic techniques were also used to investigate the distribution patterns of GBEs. We then thoroughly searched UniProt database and collected 9,387 bacterial GBEs. Through a series of identifier (ID) mapping, that is, establishment of linkages between various biological databases based on protein-centric IDs, and redundancy removal via Python programming, a final set of 9,006 GBEs was obtained (Huang et al., 2011). One hundred and forty-seven GBEs belonging to the third previously unrecognized group were highlighted in this study. They are artificially defined as possessing an approximately100 aa extension (N0 domain) ahead of the N1 domain (Figure 1). Impacts of GBE N-terminal organization on glycogen structure and bacterial fitness are also investigated theoretically in this study and are of great interests for further experimental study. In sum, through this study, we endeavored to answer the following questions by using publicly available data, which are (1) where is the N1 domain from, (2) are there any specific distribution patterns of GBE types in bacterial species from an evolutionary point of view, and (3) how many types of GBEs exist in terms of N-terminal organization?

## Materials and Methods

### GBE Sequence Collection

We initially searched UniProt database and manually reviewed a list of bacterial GBE sequences with gene name *glgB* and sequence length between 500 and 800 aa (Apweiler et al., 2017). A total of 268 GBEs were collected. Sub-species and archaeal organisms are removed through filters in UniProt database, leaving 169 bacterial GBEs in the list, which belong to 161 species due to the duplication of *glgB* in some of the strains. For details of all species and duplicated GBEs, please see Supplementary Table S1. These sequences were then used to study the domain organization and evolution of GBE in bacteria. In order to make a thorough analysis of bacterial GBE N-terminus, a total of 9,387 GBE sequences were collected from UniParc database by using following criteria: (1) taxonomy: bacteria; (2) gene name: *glgB*; (3) Protein: UniProt; and (4) Status: Active. Due to the existence of protein fragments, all sequences were further filtered and only those with length ranging from 500 to 1,000 aa were kept based on histogram analysis (96.7%). 9,083 sequences were retained. All GBEs with domain organization (CBM48, α-amylase and C-terminus) were included. 9,006 GBEs were collected, among which 5,391 GBEs have CBM48, α-amylase and C-terminus domains (Supplementary Table S2). Another 3,615 GBEs having two CBM48 domains in front of α-amylase domain were also identified (Supplementary Table S3). Among the 9,006 GBEs, those with more than 100 aa ahead of N1 domain or more than 200 aa ahead of CBM48 domain are considered as a new group of GBEs (Type 3) and marked in orange color in Supplementary Tables S2, S3, which are retrieved and recorded in a Supplementary Table S4.

### Protein Domain Distribution

**FIGURE 2:**
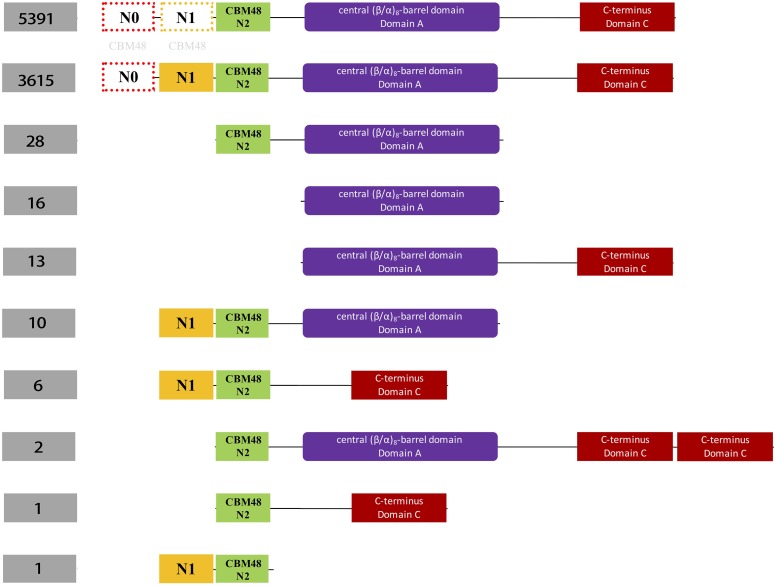
Schematic illustration of classification of 9,387 bacterial glycogen branching enzymes (GBEs) based on domain organizations from analysis of 16,712 HMMs sourced directly from PFAM database. A total of four domains are identified, which are N1 (orange), CBM48 (green). Ten groups of GBEs were identified. The number of Group 1 and Group 2 is 5,391 and 3,615, respectively. Group 1 has Type 1, Type 2, and Type 3 GBEs while Group 2 has Type 1 and Type 3 GBEs (Supplementary Tables S2, S3). The other eight groups have missing domains or unexpectedly duplicated domains (C-terminus), which will not be considered in this study. N0 and N1 domains were marked with light gray CBM48 to indicate their distant relationships.

**FIGURE 3:**
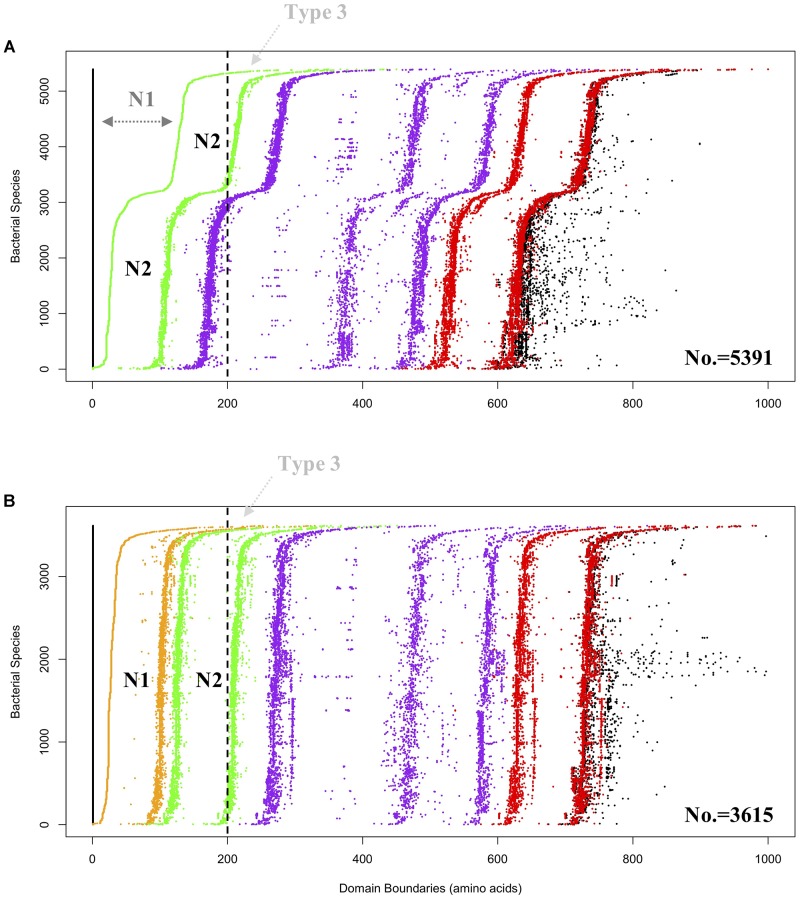
Domain visualization of bacterial GBEs in **(A)** Group 1 (5,391 sequences) and **(B)** Group 2 (3,615 sequences). Domain boundaries are denoted by dots in different colors, which are N1 (orange), CBM48 (green). Alpha-amylase (purple) and C-terminus (red). In **(A)** Group 1, dark gray N1 denoted region with dashed arrow line means that the region is too remotely related with N2 domain and cannot be detected by HMMs, which might represent a subgroup of Type 1 GBEs. Vertically dashed black line is arbitrarily drawn at 200 aa. GBEs with first detectable domain, N2 (green dots in **A**) or N1 (orange dots in **B**), starting from the vertical dashed line (black) are considered as potential Type 3 enzymes and denoted with light gray Type 3 and dashed arrow line. Boundaries of complete protein sequences are denoted in black dots. According to the domain organization, it could be inferred that both CBM48 and alpha-amylase domains experience duplications during the evolutionary process.

Hidden Markov Models (HMMs) of the three domains in GBE, that is, CBM48, α-amylase, and C-terminus, were first constructed via multiple sequence alignments (STOCKHOLM format) downloaded from online PFAM^1^ (Finn et al., 2016). HMMER3 was then used to construct HMMs via hmmbuild command (Mistry et al., 2013). Previously collected 169 sequences of GBEs were examined for their domain distributions using hmmsearch against the three HMMs. Python scripts were then used to extract domain boundaries and generate domain distributions for all sequences. Detailed boundaries of each domain in each sequence are listed in Supplementary Table S1. For the 9,083 GBEs, the corresponding sequences were scanned by 16,712 HMMs from PFAM database (E-value < 0.001). Based on presence, absence and duplication of CBM48, α-amylase, and α-amylase C-terminus domains, a total of 10 groups were identified and were ranked by quantity (Figure 2). A total of 9,006 bacterial GBEs were divided into two groups based on the number of CBM48 HMM hit(s) were visualized according to their corresponding boundaries (Figure 3). All data visualization was performed via R programming. E-values means how significant the target regions are homologous to corresponding HMMs. Thus, the smaller the E-value, the more significant the result.

### Phylogenetic Analysis and Comparison

**FIGURE 4:**
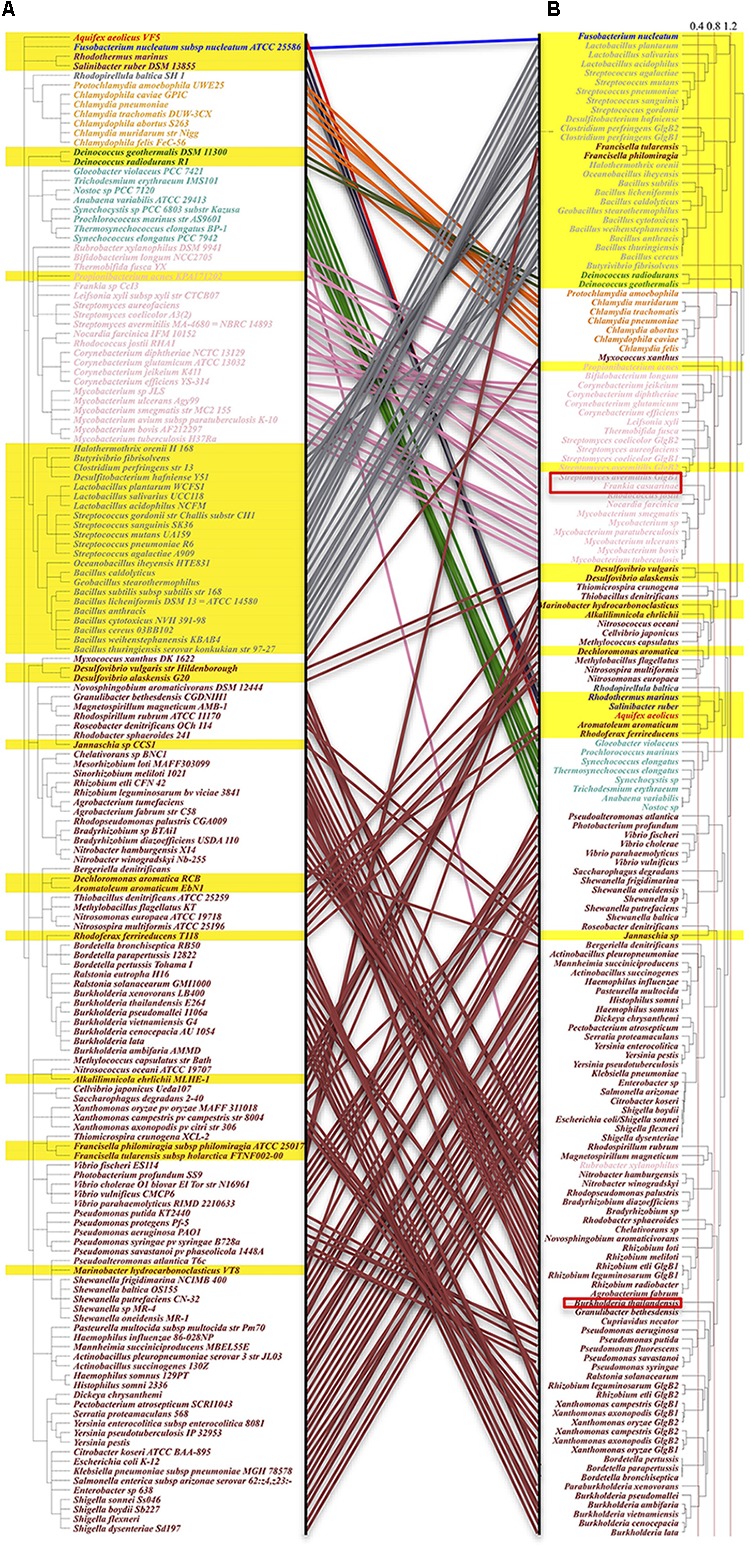
Comparison of taxonomy-based and maximum-likelihood calculated phylogenetic trees. Species orders in the phylogenetic tree are not meaningful, hence not considered in the analysis. Left: **(A)** GBEs in taxonomy-based phylogenetic tree show clustered distribution patterns. One hundred and fifty-seven species belonging to 10 phyla are analyzed, which are (from top to bottom): *Aquificae* (red), *Fusobacteria* (blue), *Bacteroidetes* (brown), *Planctomycetes* (dim gray), *Chlamydiae* (orange), *Deinococcus-Thermus* (Green), *Cyanobacteria* (light green), *Actinobacteria* (pink), *Firmicutes* (gray), and *Proteobacteria* (maroon). All Type 2 GBEs are highlighted within the range of a yellow strip. *Proteobacteria* and *Actinobacteria* are dominated with Type 1 GBE while *Chlamydiae* and *Cyanobacteria* mainly have Type 1 GBE. GBE Type 2 is identified mainly in *Firmicutes* and distributed sporadically in other phyla. Right: **(B)** Maximum-likelihood phylogenetic tree with 1,000 bootstrapping. Taxonomy- and sequence-based results are comparatively consistent at phylum level. Several GBEs from species such as *Rubrobacter xylanophilus* and *Francisella tularensis* show obviously evolutionary change that may be due to horizontal gene transfer or selective stresses for paralogs. Potential Type 3 GBEs in bacteria *Frankia casuarinae*, *Burkholderia thailandensis*, *Streptomyces avermitilis* are marked in the tree with red rectangle frameworks.

Taxonomy ID based phylogenetic tree (generated by phyloT^2^ based on NCBI taxonomy identifier) was first constructed to explore the distribution patterns of different types of GBEs at species level. Maximum likelihood (ML) phylogenetic trees based on full sequences of GBEs was constructed via MEGA7 and compared to the taxonomy tree in order to give an overview of GBE evolution (Kumar et al., 2016). ML method uses changes of amino acids or nucleotides to build a model and hypotheses were then proposed to test the model in order to find the optimal one with highest possibilities, which is a comparatively robust method for phylogenetic tree construction (Strimmer and von Haeseler, 1997). Redundant sequences were automatically removed. Interactive Tree of Life (iTOL) was used for visualization of phylogenetic trees (Letunic and Bork, 2016). The two trees were compared and corresponding bacterial species were linked through manual connection (Figure 4).

### 3D Model Construction and Superimposition of Three Types of GlgB N-Termini

**FIGURE 5:**
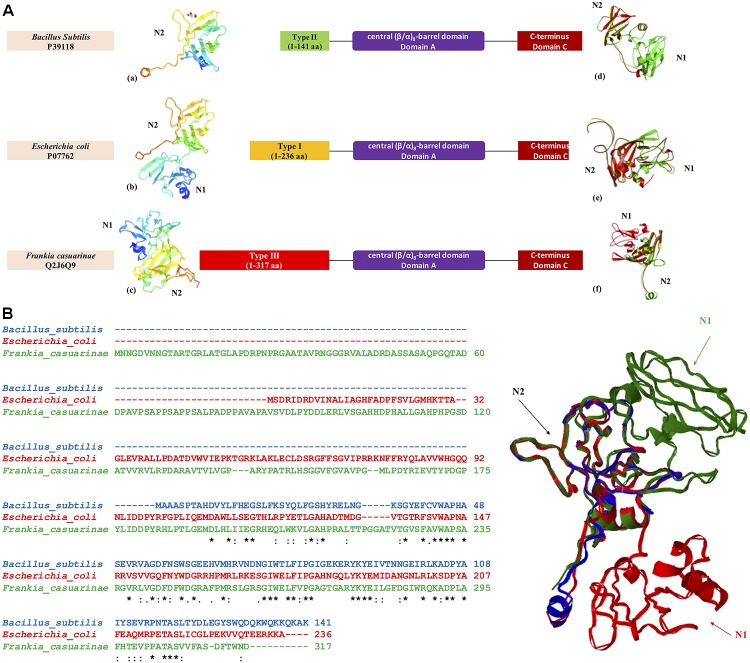
Three-dimensional structural model of GBE N-termini from three bacterial organisms *Escherichia coli* (P07762, Type 1 GBE), *Bacillus subtilis* (P39118, Type 2 GBE), and *Frankia casuarinae* (Q2J6Q9, Type 3 GBE). **(A)** N-terminal structures of GBEs (a. *Bacillus subtilis*, b. *Escherichia coli*, and c. *Frankia casuarinae*) and pair-wise superimpositions of GBE N-termini [d. *Escherichia coli* (green) and *Bacillus subtilis* (red), e. *Escherichia coli* (green) and *Frankia casuarinae* (red), and f. *Bacillus subtilis* (green) and *Frankia casuarinae* (red)]. **(B)** Multiple sequence alignments via Clustal Omega and superimposition of three bacterial GBE N-termini. Blue, red, and green ribbons represent 3D N-termini of *B. subtilis*, *E. coli*, and *F. casuarinae*, respectively. All N1 and N2 domains were specified with corresponding letters. N0 domain is failed to be constructed due to the lack of PDB template. Thus, it is not present. Star (^∗^), colon (:), and period (.) in multiple sequence alignment indicate identical, highly conservative, and semi-conservative, respectively.

GBE sequences from three bacteria *Escherichia coli* (P07762), *Bacillus subtilis* (P39118), and *Frankia casuarinae* (Q2J6Q9) were retrieved from UniProt database. N-terminus of each GBE was collected based on boundaries reported from HMMER scanning. For N-terminal boundaries and sequence details, please refer to Figure 5. 3D models of N-termini were constructed by SWISS-MODEL^3^ with default parameters [Figures 5A(a–c)]. iPBA webserver^4^ was recruited for pair-wise 3D structure superimpositions based on PDB files generated from SWISS-MODEL [Figures 5A(d–f)]. MATRAS (Markovian transition of structure evolution^5^) was then used for multiple 3D structure comparison, together with multiple sequence alignments in ClustalW format (Figure 3B).

### GBE N-Terminal Length and Glycogen ACL

Average chain lengths of glycogen in different bacteria were collected from the literature through manual check. However, data of bacterial glycogen ACLs are not sufficient in the literature. In addition, different methods were used to measure glycogen ACL. Glycogen extraction methods could also have impact on glycogen structure. Thus, there is no consistent ACL available at current stage. Corresponding GBEs were classified in terms of N-terminal lengths. The lengths of both N1 and N2 domains were listed in the table. Correlation analysis was performed to identify any potential relationships between N-terminal lengths and ACLs (Supplementary Table S5). *P*-value was greater than 0.05. No statistical significance was identified. R programming was used for all statistical analyses.

## Results

From the analysis of the manually reviewed GBEs in Supplementary Table S1 via taxonomy-based phylogenetic tree, we observed distribution patterns for GBEs at species level (Figure 4A). GBE Type 2 is present dominantly in the *Firmicutes* phylum (low G+C Gram-positive bacteria) that includes genera such as *Bacillus*, *Streptococcus*, and *Lactobacillus*, etc. Two species belonging to phylum *Bacteroidetes* also have Type 2 GBE. As for phyla of *Chlamydiae*, *Actinobacteria*, *Cyanobacteria*, and *Proteobacteria*, the former two only have Type 1 GBE while the other two have GBE Type 2 sporadically. Evolution of the GBEs is complicated due to duplication of *glgB* gene in some bacterial genomes, such as one Type 1 GBE (*Escherichia coli*), one Type 2 GBE (*Deinococcus radiodurans*), two different types of GBEs (*Streptomyces avermitilis*), two Type 1 GBEs (*Rhizobium etli*), and two Type 2 GBEs (*Clostridium perfringens*). There are also bacterial species with multiple duplications of GBEs that will not discussed here. Taxonomy- and ML-based phylogenetic trees were compared in order to identify how evolutionary pathways differ for GBEs at the species (Figure 4A) and sequence levels (Figure 4B). According to the study, phyla *Chlamydiae*, *Cyanobacteria*, and *Firmicutes* show consistent clustering patterns, although the orders change. GBEs of *Firmicutes* and *Chlamydiae* are more closely related while *Cyanobacteria* is clustered together with *Proteobacteria*. As for *Actinobacteria*, GBE in a single species *Rubrobacter xylanophilus* is more related with *Proteobacteria* phylum at sequence level while all other GBEs in *Actinobacteria* are grouped together. GBEs in *Francisella philomiragia*, *Francisella tularensis*, and *Myxococcus xanthus* are distinguished from other GBEs in *Proteobacteria* phylum based on sequence analysis, which may provide novel clues for GBE evolution in bacteria.

HMM-based sequence analysis showed that N-terminal lengths of bacterial GBEs ahead of CBM48 were variable and changed continuously from 5 to 229 aa (Supplementary Table S1). By following previous grouping rules, GBEs were divided into two groups. A new third group with 100 or more amino acids in front of N1 and CBM48 domains has never been mentioned or experimentally studied. Further investigation of 9,387 bacterial GBEs showed a similar trend of N-terminal variability. Based on 16,712 HMM-scanned results, we extracted all boundary information for domains in GBE sequences and visualized the data using Python and R scripts. According to the domain organization, all GBEs are classified into 10 groups, among which Group 1 have 5,391 sequences (Supplementary Table S2) while Group 2 has 3,615 sequences (Supplementary Table S3) while all other eight groups have missing or unexpectedly duplicated domains (Figure 2). The two major groups of GBEs were then visualized in terms of domain boundaries (Figure 3). Apparent displacement and duplication of the CBM48 domain was observed. Based on N-terminal length, these GBEs were artificially divided into three groups. Analysis of 169 bacterial GBE sequences showed that three GBEs (UniProt Accession Numbers: Q2J6Q9, Q2T6R3, Q82JF0) from *Frankia casuarinae*, *Burkholderia thailandensis*, and *Streptomyces avermitilis* are 100 aa longer at N-terminus than Type 1 GBE. Similar to the 169 manually reviewed GBEs, there are 147 GBEs with a previously unidentified third GBE Type in the 9006 bacterial GBEs (Supplementary Table S4). Due to the selection pressure, N-terminal sequences change vastly. Thus, division of GBEs based on N-terminal organization was applied. For Type 1 GBE, HMM-based homologous searches of N-terminal domains revealed that N1 and N2 shared variable levels of similarity based on E-value comparisons (Supplementary Table S3). Here, E-value is generated during HMM search and means how likely the target region in a sequence is a homolog of the HMM. Higher and similar E-value between N1 and N2 indicates a more recent duplication of N2 domain. On the other hand, lower E-value indicates a remote relationship between the two domains and there is a vast change of amino acid sequences. Thus, except for previously reported Type 1 and 2 GBEs, we identified 147 GBEs with more than 200 aa ahead of CBM48 domains that might form a potential new type group, Type 3 GBEs (Supplementary Table S4).

In order to achieve a better understanding of GBEs, we also constructed and compared three-dimensional structures of the three types of N-termini sourced from *Escherichia coli* (P07762), *Bacillus subtilis* (P39118), and *Frankia casuarinae* (Q2J6Q9). Individual 3D models and pair-wise superimpositions showed that Type 1 and 2 GBE N-termini were similar while type 3 GBE N-termini were complex and differed from the other two types. Superimposition of the three N-termini found that they shared a common fragment CBM48. Due to the lack of N0 template in PDB database, for all N-terminal models, only N1 and N2 are constructed.

Previously, experimental studies showed that N-terminal organization of GBEs linked with glycogen ACL (Jo et al., 2015; Wang et al., 2015). Since GBE N-terminus can be divided into distinct groups, we also investigated the relationships between GBE types and ACL of bacterial glycogen through literature mining and correlation analysis. A total of 18 bacterial species were collected. Sequence analysis showed that 10 GBEs belong to Type 1 and the other 8 belong to Type 2. Student’s *t*-test analysis showed no statistically significant difference between the two groups (*P* > 0.05), although glycogen in species with Type 1 GBEs (11.56 d.p.) had moderately shorter ACL than Type 2 GBEs (12.38 d.p.) (Supplementary Table S5).

## Discussion

In the *Escherichia coli* genome, the five essential genes for glycogen metabolism are organized into a single operon *glgBXCAP* with a suboperonic promoter in *glgC* gene (Montero et al., 2011). Although several bacteria, such as *Salmonella typhi*, *Yesinia pestis*, and *Shigella flexner*i, share the same gene organization for glycogen metabolism as *E. coli*, the operon structure is not widely conserved. Missing, duplicated, and/or external genes occur in their operons compared with the canonical arrangement of genes in *E. coli*, reflecting the complexities of glycogen metabolism and its wide connections with other pathways, such as glucose, trehalose and maltose, etc., in bacteria (Cho et al., 2008; Almagro et al., 2015). Recently, phylogenetic study tracked the last common ancestor for the *glgBXCAP*-like gene organization between sister orders of *Enterobacteriales* and *Pasteurellales* (Almagro et al., 2015). How these organizations are able to influence glycogen structure is currently unknown and will require further research. Beyond *glgBXCAP*, studies have also found a variety of enzymes involved in glycogen metabolism. A Keio collection based screening of glycogen related non-essential genes in *E. coli* identified 35 glycogen-excess and 30 glycogen-deficient mutants, respectively (Eydallin et al., 2007). It is noteworthy that GlgS in this study is not identified as a glycogen related enzyme because the *ΔglgS* deletion mutant accumulates normal glycogen content, which contradicts previous studies and indicates a complex role for this gene in bacterial glycogen metabolism (Hengge-Aronis and Fischer, 1992; Eydallin et al., 2007). Another distinct glycogen-related enzyme maltosyltransferase GlgE uses trehalose as a precursor in bacteria for glycogen synthesis and has been reported to strongly correlate with GlgB pathway through comparative genomic analysis (Chandra et al., 2011). Further studies show that GlgE has a linkage with the synthesis of the more condensed and short-chained glycogen-like α-glucan in outer capsule of *Mycobacterium tuberculosis* (Bornemann, 2016). However, compared with 59% representation of GlgB in 1,202 sequenced bacterial genomes, only 14% GlgE was identified (Chandra et al., 2011; Wang and Wise, 2011).

The durable energy storage mechanism (DESM) hypothesis correlates glycogen ACL with bacterial durability (Wang and Wise, 2011). According to a series of studies, GBE N-terminus has impact on glycogen ACL (Binderup et al., 2002; Devillers et al., 2003; Jo et al., 2015; Wang et al., 2015). Thus, investigation of N-termini of GBEs in bacteria has potential application for indicating the structure of bacterial glycogen. Analysis based on remote sequence homologies predicted that N1 domain originates from duplication of N2 domain and both of the two N-terminal domains have a immunoglobulin-type structure (Leggio et al., 2002). Crystal structure analysis of GlgB in *M. tuberculosis* H37Rv also showed that N1 and N2 beta-sandwich structures superimpose well with each other (Pal et al., 2010). As for C-terminus, no evidence correlates it with substrate specificity and chain transfer pattern (Palomo et al., 2009). Previous studies only used a small set of bacterial GlgB to do the phylogenetic analysis for N1 domain (Leggio et al., 2002; Jo et al., 2015). Our study, based on 9,387 bacterial GBEs, confirmed that 9,006 sequences have non-redundant canonical domain organization, which forms Type 1 and Type 2 GBEs with possible Type 3 GBE unrevealed. Lengths of sequences before the CBM48 domain vary. An apparent domain displacement can be observed, which indicates that CBM48 duplication and random mutation interact to affect the variability of GBE N-termini (Figure 3). Due to the variability of E-values, we can also conclude that N1 domain changes more quickly than N2 domain. Less conservation existed between N1 domains. In 5,391 GBEs, only a single CBM48 is identified in each protein based on HMM search. However, in 3,615 GBEs, all of them have two significant CBM48 hits at the N-terminus and the two hits share certain degree of similarity, indicating higher level of similarities between N1 and N2 in terms of sequence homology in this group (Supplementary Table S3) (Binderup et al., 2002; Leggio et al., 2002). Thus, there are probably two subgroups of Type 1 GBEs: (1) those with two identified homologs to CBM48 and (2) those with a single identified CBM48 domain with a less similar and non-homologous N1 domain. During the analysis of N-terminal lengths and GBEs types, we also spotted a special group with more than 200 aa in front of the CBM48 domain. Considering that N1 domain is duplicated from CBM48 domain, the extended N-terminus might harbor a putative N0 domain and could contribute to transfer extremely short chains during glycogen synthesis. That is, like the N1 domain, N0 domain could also be distant relative to the standard CBM48 domain found in Pfam. However, no evidence is available for the functions and structure of long N-terminal GBEs at current stage. More theoretical and experimental studies are needed to better understand the relationships between the structure and functions. It is also worthy of mentioning that there are apparently three lines of purple dots indicating coexistence of long (approximately 300 residues), medium (approximately 200 residues) and short (approximately 100 residues) Alpha-amylase domains (Figure 3). Overall, the shorter, Type 2 GBEs are predominantly found in Gram-positive species, while the longer Type 1 GBEs are found in Gram-negative species, according to the preliminary analysis of 169 bacterial reference GBEs.

In the two phylogenetic trees based on taxonomy identifiers and maximum likelihood, we observed clustered patterns that are consistent between the evolution of species and GlgB genes, although several GBEs are grouped together with distantly related species, which may indicate horizontal gene transfer, high selective pressure or convergent evolution (Suzuki and Suzuki, 2016). Explanations are required for a better understanding of the evolution of GlgB in bacteria. According to the phylogenetic analysis, it is also possible that some Type 2 GlgB genes obtained N2 duplication first to become Type 1 GlgB and then experienced N1 depletion (GlgB2 in *Streptomyces avermitilis*) or Type 1 GBE experienced N1 depletion (GlgB in *Jannaschia* sp.). Thus, the presence and absence of N1 domain in GlgB is probably reversible and determined by bacterial living environment. In fact, domain duplication and loss happen during protein evolution comparatively frequently. Analysis of the domain organization of our 9083 bacterial GBEs showed that all three domains can have be duplicated or truncated (Figure 2), which might have evolutionary advantage in specific niches. Although several studies suggested the role of GlgB N-terminus in glycogen structure for short chain preference, it does not exclude alternative options. The observed pattern shift might be caused by changed spatial structure and reduced catalytic activity of GlgB due to N1 truncation (Feng et al., 2015). It may also be caused by the imbalance among glycogen synthesis and degradation enzymes due to GlgB truncation (Wilson et al., 2010; Goh and Klaenhammer, 2013). After all, the full-length GlgB is the optimized result of long-term evolution and any change to GlgB could lead to abnormal synthesis of glycogen. During the analysis of 169 manually reviewed sequences, we also found that the bacterial set includes several multi-copy GBEs. Studies show that multi-copy GBEs contribute to bacterial physiology and glycogen metabolism in a differentially spatial and temporal manner (Bruton et al., 1995; Schneider et al., 2000; Rueda et al., 2001). Thus, it is also worth further experimental study in terms of their classifications, distributions and functions to better understand glycogen metabolism in bacteria.

## Conclusion

In this study, we systematically investigated GBEs in thousands of bacterial species from the perspective of sequence evolution and domain structures. Phylogenetic comparison of taxonomy- and ML-based trees showed that most of bacterial GBEs evolve consistently at species and sequence level due to similar cluster patterns, although there are several exceptions, which may be caused by other forces such as horizontal gene transfer or convergent evolution (Suzuki and Suzuki, 2016). Multi-copy GBEs also exist to fulfill bacterial physiological requirements at differential development phases (Bruton et al., 1995; Schneider et al., 2000; Rueda et al., 2001). So far, GBEs with classic domain organization are divided into Type 1 and 2 groups (Hilden et al., 2000; Leggio et al., 2002; Devillers et al., 2003; Palomo et al., 2009; Pal et al., 2010; Jo et al., 2015; Wang et al., 2015). In addition, we reported the potential existence of Type 3 GBEs, that is, GBE with more than 100 aa ahead of N1 domain. Several experimental and theoretical studies confirmed that N-terminal length is related to glycogen ACL and short ACL glycogen contributes to bacterial durability (Wang and Wise, 2011; Wang et al., 2015). However, correlation analysis in this study did not show statistical significance between GBE types and glycogen primary structure (ACL), in spite that moderate difference was observed. A possible reason for non-correlation is that the analysis used insufficient amount of data from a variety of sources. Thus, future studies should probably focus on applying standardized glycogen extraction and structure characterization techniques in more bacterial species. Considering the importance of GBE N-terminus for glycogen structure, it is worth to further investigate the functions of the extended N-terminus in type 3 GBE and compare the results with these from Type 1 and 2 GBEs. For example, *in situ* expression of three types of GBEs in a model microorganism and compare glycogen structures, which may give a hint on how the types of N-terminus correlate with glycogen ACLs. Due to the high variability and comparative uniqueness of N-terminal domain in bacterial GBEs, it may also serve as a drug target for weakening bacterial persistence in macrophages due to the potential linkage between glycogen structure and bacterial viability in terms of energy supply. In order to draw a clear picture of GBE functions in glycogen structure, more experimental studies are required.

## Author Contributions

LW and MW conceived the core ideas and designed the experiments. LW, QL, JA, XW, JH, and JY performed the experiments. LW, XW, CM, and XC analyzed the data. XC and DT contributed analysis tools. LW, QL, JA, JH, and JY prepared the figures and/or tables. LW, JH, MW, and DT drafted the work or revised it critically for important content. All the authors approved the final draft of the manuscript submitted for review and publication.

## Conflict of Interest Statement

The authors declare that the research was conducted in the absence of any commercial or financial relationships that could be construed as a potential conflict of interest.

## Footnotes

1 http://pfam.xfam.org/database

2 http://phylot.biobyte.de/

3 https://swissmodel.expasy.org/

4 http://www.dsimb.inserm.fr/dsimb_tools/ipba/index.php

5 http://strcomp.protein.osaka-u.ac.jp/matras/matras_multi.html
