# Supplementary material for: Structure and Evolution of Glycogen Branching Enzyme N-Termini From Bacteria
Source: Front Microbiol. 2019 Jan 14;9:3354. doi: 10.3389/fmicb.2018.03354 (PMC6339891; doi:10.3389/fmicb.2018.03354)
Supplement: Supplementary file 5 [file Table_5.DOCX]

**Supplementary Table S5** Correlational analysis of GBE types and glycogen average chain length in a set of bacteria. Lengths of N1 and N2 domains are presented. Unpaired Student’s *t*-test was performed to calculate the correlation of GBE types and glycogen average chain lengths (ACLs) by comparing ACLs of Type 1 and Type 2 GBEs. UniProt_AC is identifier of protein item in UniProt database. No statistical significance was found (P>0.05).

| **Bacteria** | **GBE Types** | **N1** | **N2** | **ACL** | **UniProt_AC** | **Ref** |
| --- | --- | --- | --- | --- | --- | --- |
| *Aerobacter aerogenes* | 1 | 123 | 84 | 13 | A0A0H3FND8 | ([1](#_ENREF_1)) |
| *Agrobacterium tumefaciens* | 1 | 132 | 85 | 13 | Q8U8L4 | ([2](#_ENREF_2)) |
| *Allochromatium vinosum* | 1 | 124 | 84 | 11 | D3RQ23 | ([1](#_ENREF_1)) |
| *Clostridium botulinum* | 1 | 103 | 86 | 17 | B2TR29 | ([1](#_ENREF_1)) |
| *Escherichia coli* | 1 | 123 | 84 | 12 | P07762 | ([1](#_ENREF_1)) |
| *Klebsiella pneumoniae* | 1 | 123 | 84 | 11.6 | A6TF51 | ([1](#_ENREF_1)) |
| *Mycobacterium tuberculosis* | 1 | 128 | 88 | 11 | P9WN45 | ([2](#_ENREF_2)) |
| *Sphaerotilus natans* | 1 | 137 | 84 | 11.5 | A0A059KQQ1 | ([1](#_ENREF_1)) |
| *Synechocystis sp. PCC6803* | 1 | 127 | 83 | 7.5 | P52981 | ([2](#_ENREF_2)) |
| *Vibrio vulnificus* | 1 | 113 | 83 | 8 | Q7MG90 | ([3](#_ENREF_3)) |
| *Aquifex aeolicus* | 2 | - | 84 | 10 | O66936 | ([4](#_ENREF_4)) |
| *Bacillus megaterium* | 2 | - | 84 | 10.5 | G2RU45 | ([1](#_ENREF_1)) |
| *Deinococcus geothermalis* | 2 | - | 83 | 7 | Q1IZQ3 | ([5](#_ENREF_5)) |
| *Deinococcus radiodurans* | 2 | - | 83 | 7 | Q9RTB7 | ([5](#_ENREF_5)) |
| *Geobacillus stearothermophilus* | 2 | - | 84 | 21 | P30538 | ([1](#_ENREF_1)) |
| *Prevotella ruminicola* | 2 | - | 88 | 8 | D8DYY0 | ([1](#_ENREF_1)) |
| *Selenomonas ruminantium* | 2 | - | 85 | 23.5 | I0GN11 | ([1](#_ENREF_1)) |
| *Streptococcus mitis* | 2 | - | 83 | 12 | F9LXZ4 | ([1](#_ENREF_1)) |

**Reference**

1. **White J.** 2014. Manupulation of Storage Polysaccharides in Microorganism. The University of Edinburgh, Edinburgh.

2. **Martínez García M.** 2017. Functional carbohydrates from the red microalga Galdieria sulphuraria. University of Groningen, Groningen.

3. **Jo HJ, Park S, Jeong HG, Kim JW, Park JT.** 2015. Vibrio vulnificus glycogen branching enzyme preferentially transfers very short chains: N1 domain determines the chain length transferred. FEBS letters **589:**1089-1094.

4. **Kajiura H, Takata H, Akiyama T, Katukani R, Furuyashiki T, Kojima I, Harui T, Kuriki T.** 2011. In vitro synthesis of glycogen: the structure, properties, and physiological function of enzymatically-synthesized glycogen. Biologia **66:**8.

5. **Palomo M, Kralj S, van der Maarel MJEC, Dijkhuizen L.** 2009. The unique branching patterns of Deinococcus glycogen branching enzymes are determined by their N-terminal domains. Applied and environmental microbiology **75:**1355-1362.
